# Influence of antibacterial surface treatment on dental implants on cell viability: A systematic review

**DOI:** 10.1016/j.heliyon.2023.e13693

**Published:** 2023-02-16

**Authors:** Renan Leonardi de Oliveira Rigotti, Juliana Dias Corpa Tardelli, Andréa Cândido dos Reis

**Affiliations:** Department of Dental Materials and Prosthesis; School of Dentistry of Ribeirão Preto, University of São Paulo (USP), Ribeirão Preto, SP, Brazil

**Keywords:** Dental implants, surface treatment, Cell viability, Bacteria, Peri-implantitis

## Abstract

There is no consensus in the literature about the best non-cytotoxic antibacterial surface treatment for dental implants. Critically evaluate the existing literature and answer the question: “which surface treatment for dental implants made of titanium and its alloys has the greatest non-cytotoxic antibacterial activity for osteoblastic cells?” This systematic review was registered in the Open Science Framework (osf.io/8fq6p) and followed the Preferred Reporting Items for Systematic Review and Meta-analysis Protocols. The search strategy was applied to four databases. Articles were selected that evaluated in both studies the properties of 1) antibacterial activity and 2) cytotoxicity on osteoblastic cells of titanium and their alloy dental implants when treated superficially. Systematic reviews, book chapters, observational studies, case reports, articles that studied non-dental implants, and articles that evaluated only the development of surface treatment were excluded. The Joanna Briggs Institute, a quasi-experimental study assessment tool, was adapted to assess the risk of bias. The search strategy found 1178 articles in the databases after removing duplicates in EndNote Web, resulting in 1011 articles to be evaluated by title and abstract, of which 21 were selected for full reading, of which 12 were included by eligibility criteria, and nine were excluded. Quantitative synthesis could not be performed due to the heterogeneity of the data (surface treatment, antibacterial assay, bacteria strain, cell viability assay, and cell type). Risk of bias assessment showed that ten studies were classified as low risk and two studies as moderate risk. The evaluated literature allowed us to conclude that: 1) The literature surveyed did not allow answering the question due to the heterogeneity of the studies; 2) Ten of the 12 studies evaluated presented surface treatments with non-cytotoxic antibacterial activity; 3) Adding nanomaterials, QPEI, BG, and CS, reduce the chances of bacterial resistance by controlling their adhesion by electrical forces.

## 1 Introduction

The development of pro-osteogenic and antibacterial bioactive surfaces is promising for success since implant-supported oral rehabilitations are susceptible to challenging oral and systemic patient conditions, which can hinder the integration of the biomaterial into bone tissue [1,2]. Lindhe e Meyle [3] reported that the risk of peri-implant mucositis is around 80% and peri-implantitis 28–56% after implant surgeries. Thus, the microbial challenge facing the surgery to install dental implants leads to proposals to understand the microbiological, bio-physical-chemical, and structural factors involved in the implant/bone interaction to prevent peri-implant infections and their deleterious effects on treatment success are the focus of study [[4], [5], [6], [7], [8]].

Titanium alloys (Ti) are the materials of choice for dental implants due to their biocompatibility, excellent mechanical and physical-chemical properties, and chemical stability [5], [6], [7], [9], [10], [11], [12], [13], [14], [15]. However, the most widely used alloy, Ti–6Al–4V, is the subject of discussions, as its elements Al and V are considered neurotoxic [7,[16], [17], [18], [19], [20], [21], [22]]. Therefore, beta titanium alloys have become promising in recent years, as they incorporate non-cytotoxic chemical elements such as molybdenum (Mb), tantalum (Ta), zirconium (Zr) and niobium (Nb). As its modulus of elasticity is closer to the bone, adaptive proximal bone remodeling (stress shielding) can be avoided, as it allows for an adequate distribution of the forces to which the implant is subjected [7,16,17,[23], [24], [25], [26]].

One of the main causes of implant failure is a peri-implant infection caused by specific microbiota, which is associated with periodontitis. *Streptococcus* spp. is the pioneer in the colonization of the surgical site and peri-implantitis microbiota and includes *Fusobacterium* spp.*, Prevotella intermedia, Actinobacillus actinomycetemcomitans, Peptostreptococcus micros, Campylobacter rectus, Capnocytophaga rectus, Capnocytophaga* spp. and *Porphyromonas gingivalis* [10,27]; associated with high rates of patient dissatisfaction and cost of rehabilitation treatment. In addition, peri-implant infection is more predisposed in smokers and patients with chronic periodontal disease [9,10,13,14,[27], [28], [29]].

The literature presents surface treatments classified as antibacterial by adding antibacterial ions, drugs, and nanomaterials, and non-antibacterial treatments can potentially reduce bacterial adhesion by reducing the favorable area for bacterial colonization [7,8,27,[30], [31], [32], [33], [34], [35], [36], [37], [38]]. An et al. [39] demonstrated that the anodizing treatment by increasing the diameter of the TiO2 nanotube reduced bacterial activity. The Kreve and Reis 2021 [40] review infers that the regulation of the electrostatic condition of the surface is a promising treatment for regulating bacterial adhesion on titanium surfaces through long-range forces that dictate attraction or repulsion. Treatments without the addition of antibacterial have the advantage of reducing the risk of bacterial resistance by regulating adhesion through physicochemical and electrostatic changes.

Antibacterial surface treatments include the addition of antibacterial ions such as silver (Ag), copper (Cu), and zinc (Zn) that act by disrupting the bacterial cell wall and inhibiting the activity of respiratory enzymes and DNA replication [[41], [42], [43], [44], [45], [46], [47], [48]]. The addition of antibiotics to synthetic poly (adipic anhydride) ((C6H8O3)n) (PADA) and poly(lactic acid-co-glycolic acid) (PLGA) and natural chitosan (CS) polymers allows direct delivery of the drug to the site desired in lower doses if administered orally. However, it is a complex technique because it involves more than one step to be carried out and presents challenges such as determining the concentration of antibiotic that is antibacterial and non-cytotoxic [7,33].

In addition to the surface mentioned above treatments, the addition of antibacterial nanomaterials has shown promise. Thus, bioactive glass (BG), in addition to being known for its osteoinductive capacity, also has antibacterial activity by modulating the rate of ion release, which influences the pH and osmolarity by changing the physiological conditions of the implantation bed [35,49]. The incorporation of polyethyleneimine quaternary ammonium nanoparticles (QPEI) promotes antibacterial activity by electrostatic interaction with bacterial cells [31]. The synthesis of chimeric peptides and their application on titanium surfaces demonstrates antibacterial activity also by electrostatic interaction and by disruption of the bacterial cell membrane and inhibition of RNA replication [32].

Many superficial treatments are available in the literature, with different mechanisms of action and proposals. However, there is no consensus in the literature about the best non-cytotoxic antibacterial surface treatment for titanium dental implants and their alloys. Therefore, the objective of this systematic review was to critically evaluate the existing literature and answer the question: “which surface treatment for dental implants made of titanium and its alloys has the greatest non-cytotoxic antibacterial activity for osteoblastic cells?”

## 2 Material and methods

The systematic review was registered in Open Science Framework (osf.io/5jenw) and followed the Preferred Reporting Items for Systematic Review and Meta-Analyses Protocols [50,51]. The PICOS was structured according to the focus question, “which surface treatment for dental implants made of titanium and its alloys has the greatest non-cytotoxic antibacterial activity for osteoblastic cells?” being: Population = titanium and their alloys surfaces for dental implants; Intervention = any surface treatment; Comparison = control group; Outcomes = antibacterial activity and cytotoxicity for osteoblast cells; and Study design = *in vitro* studies.

Articles were selected to compose this systematic review from *in vitro* experimental studies that evaluated the antibacterial activity and cytotoxicity for osteoblastic cells of surface treatments applied on titanium surfaces and their alloys for dental implants. In addition, were excluded 1) Did not study dental implants, 2) Did not evaluate cell viability on osteoblast cells, 3) Did not study titanium and their alloys, 4) Did not evaluate the antibacterial activity of surface treatment, 5) Full-text article not available. Also, the eligibility criteria as described in Table 1.

**Table 1:** Eligibility criteria.

Eligibility Criteria	
Inclusion	Exclusion
Articles that evaluated in the same study both properties 1) antibacterial activity and 2) cytotoxicity on osteoblastic cells of titanium and their alloys dental implants when treated superficially.	Systematic reviews, book chapters, observational studies, and case reports;
	Articles that studied non-dental implants;
	Articles that evaluated only the development of surface treatment.

The search strategy was applied in the databases Embase, PubMed, Science Direct, and Scopus on November 17th, 2022, without time and language restrictions (Appendix 1). The citation manager was inserted in the EndNote Web to save and remove duplicates after the citation manager was inserted in the Rayyan web app to analyze the titles and abstracts in the first phase.

The selection process was realized in two phases. In the first phase, the reviereviewer's R.LO.R and J.D.C.T assessment independently the titles and abstracts to find articles that meet eligibility criteria. In the second phase, the articles selected in the first phase were read in full to select articles for this review. The coordinator, A.C.R., solved the doubts in the consensus meeting.

The reviewers independently tabulated data in a Microsoft Excel spreadsheet according to Author, year; Population (alloy, groups); Intervention (surface treatment); Comparison (control); Outcome for antibacterial activity (assay, bacteria, and result); the outcome for cell viability (assay, cell, and result) and, Conclusion were detailed in Table 2, Table 3.

**Table 2:** Characteristics of studies that evaluated antibacterial surface treatment.

Author, year	Intervention	Comparison			Outcome – Antibacterial activity assay		Outcome – Cell viability assay			Conclusion
	Groups	Surface treatment	Control	Essay	Bacteria	Result	Essay	Cell	Result	
Kazek-Kęsik et al., 2019 [33]	Ti–15Mo Bacterial assay G4 = Ti–15Mo/PEO/PLGA/AMX; Control = 30 μg of amoxicillin. Osteoblast assay G1 = Ti–15Mo G2 = Ti–15Mo/PEO; G3 = Ti–15Mo/PEO/PLGA; G4 = Ti–15Mo/PEO/PLGA/AMX.	dip-coating technique	Bacterial assay 30 μg of amoxicillin. Osteoblast assay Ti–15Mo	inhibition zones	*S. aureus* and S. epidermidis	*S. aureus* G4 = 22–24 mm; Control = 32 mm. S. epidermidis G4 = 30–34 mm; Control = 35 mm.	Alamar Blue	MG-63	All were biocompatible. But there was a greater proliferation in the sequence. G1>G2>G3>G4. With a significant difference of p < 0.01 compared to G3 and G4	The addition of AMX to the coating allows it to present the ability to inhibit the evaluated bacteria in 1h. However, it reduces osteoblastic viability.
Leśniak-Ziółkowska et al., 2020 [7]	Ti–2Ta–3Zr–36Nb Bacterial assay G1 = Ti–2Ta–3Zr–36Nb; G2 = Ti–2Ta–3Zr–36Nb/PEO; G3 = Ti–2Ta–3Zr–36Nb/PEO/PADA; G4 = Ti–2Ta–3Zr–36Nb/PEO/PADA/AMX; G5 = Ti–2Ta–3Zr–36Nb/PEO/PADA/CEF; G6 = Ti–2Ta–3Zr–36Nb/PEO/PADA/VAN. Osteoblast assay G1 = Ti–2Ta–3Zr–36Nb; G2 = Ti–2Ta–3Zr–36Nb/PEO; G4 = Ti–2Ta–3Zr–36Nb/PEO/PADA/AMX; G5 = Ti–2Ta–3Zr–36Nb/PEO/PADA/CEF; G6 = Ti–2Ta–3Zr–36Nb/PEO/PADA/VAN.	dip-coating technique	Bacterial assay and Osteoblast assay Ti–2Ta–3Zr–36Nb	inhibition zones	Reference *S. aureus* and clinical *S. aureus*	Reference *S. aureus* G1 = 5 mm; G2 = 5 mm; G3 = 5 mm; G4 = 30 mm; G5 = 15 mm; G6 = 12 mm. clinical *S. aureus* G1 = 5 mm; G2 = 5 mm; G3 = 5 mm; G4 = 18 mm; G5 = 24 mm; G6 = 16 mm.	Alamar Blue	MG-63	There was no significant difference in cell viability. When observing the reduction in viability, it was: G1 = 0.4%; G2 = 17.2%; G4 = 22%; G5 = 18.5%; G6 = 21.6%.	All coatings evaluated with antibiotics were cytocompatible and inhibited the formation of bacterial colonies, with AMX being the best.
López-Ortega et al., 2022 [34]	Ti–20Nb–20Zr–4Ta Bacterial assay And Osteoblast assay G1 = Ti–20Nb–20Zr–4Ta G2 = Ti–20Nb–20Zr–4Ta/PEO/Ca + P G3 = Ti–20Nb–20Zr–4Ta/PEO/Ca + P/Ag	PEO	Bacterial assay and Osteoblast assay: Ti–20Nb–20Zr–4Ta	CFU	*E. coli*	G3>G2 = G1 The G3 group showed a significant reduction in bacterial activity. However, it is noteworthy that the p-value was not reported.	CCK-8	MC3T3-1	G2>G3>G1 p < 0.001 All coatings were biocompatible	All groups were biocompatible, and G2 showed a significant reduction in bacterial activity.
Mokhtari et al., 2018 [35]	Bacterial assay And Osteoblast assay Ti G1 = Ti; G2 = Ti/58S-BG-CS; G3 = TNT; G4 = TNT/CS; G5 = TNT/58S-BG-CS	Dip-coating	Bacterial assay and Osteoblast assay Ti and TNT	CFU	*E. coli* and *S. aureus*	G5>G4>G3>G2>G1 G5: *E. coli* = 89 ± 3% *S. aureus* = 71 ± 3% p < 0.05	MTT	MG-63	G5>G4>G3> G2>G1 p < 0.05	The TNT/58S-BG-QS coating has improved antibacterial and osteogenic activity.
Norowski et al., 2011 [27]	Bacterial assay And Osteoblast assay Ti G1 = Ti; G2 = Ti/CS; G3 = Ti/CS/20%TC; G4 = Ti/CS/0,02%CHX.	Silane reactions	Bacterial assay Positive control: 200 μL sterile media. Negative controls: 50 μL sterile broth. Osteoblast assay Chitosan (control for chlorhexidine and tetracycline)	Turbidimetric assays	A. actinomycetemcomitans and S. epidermidis.	A. actinomycetemcomitans G3 = 94%; G4 = 56-0%. S. epidermidis G3 = 99%; G4 = 100-95%. No growth inhibition for G1 and G2.	MTT	HEPM	All were biocompatible. There was no significant difference in viability. It should be noted that CHX caused a 58% reduction in da 1.	The coatings showed antibacterial activity and were not cytotoxic.
Pistilli et al., 2018 [36]	Bacterial assay And Osteoblast assay Ti G1 = Control Ti/MAC G2 = Control Ti/AE G3 = Control Ti/ZrN G4 = Ti/MAC G5 = Ti/AE G6 = Ti/ZrN	RF-PECVD	Bacterial assay and Osteoblast assay Control Ti/MAC, Ti/AE and Ti/ZrN	CFU	sputum sample obtained from one healthy volunteer	G6>G5>G4 p = 0.0167	CLSM	MC3T3-E1	G5>G6>G4 p = 0.010	G6 demonstrated non-cytotoxic antibacterial activity.
Song et al., 2022 [30]	Bacterial assay and Osteoblast assay Ti EPD in Direct Current = G1 = Ti/CS G2 = Ti/CS/CEF G3 = Ti/CS/Ca3(PO4)2 G4 = Ti/CS/Ca3(PO4)2/CEF EPD in Pulsed Current = G5 = Ti/CS G6 = Ti/CS/CEF G7 = Ti/CS/Ca3(PO4)2 G8 = Ti/CS/Ca3(PO4)2/CEF	EPD	Bacterial assay Control and Ti Osteoblast assay Ti and alkali-treated Ti.	optical density	*Staphylococcus aureus*	G6 (97,9%)>G2 (97,5%)>G4 (96,1%)>G8 (80,6%)	optical density	MG-63	TiA > G8>Ti > G6 p < 0.001 entre TiA e G6	Only G8 showed non-cytotoxic antibacterial activity. The other coatings, despite being antibacterial, were cytotoxic.
Sun et al., 2019 [31]	Bacterial assay And Osteoblast assay Ti G1 = Ti G2 = Ti/ALEN G3 = Ti/QPEI G4 = Ti/QPEI-ALEN	post-functionalization of polymer brushes	Bacterial assay and Osteoblast assay Ti	live/dead staining	*S. aureus*	G4>G3>G2>G1 G1 not shows antibacterial activity p < 0.0001 entre G1 e G4	CLSM	MC3T3-E1	G1 = G2 = G4>G3 G3 showed significant cytotoxicity. However, it is noteworthy that the p-value was not reported	The use of ALEN associated with QPEI promoted non-cytotoxic antimicrobial activity. However, the use of only QPEI demonstrated cytotoxicity

AE, dual etching; ALEN, alendronate; Ag, silver; AMX, amoxicillin; Ca, calcium; Ca3(PO4)2, calcium phosphate; CCK-8, cell count kit 8; CEF, cephalexin; CFU, colony forming units; CS, chitosan; EPD, electrophoretic deposition; HEPM, derived from mesenchymal cells from the human embryo; MAC, machined; MTT, tetrazol microculture; P, phosphor; PADA, poly (adipic anhydride) ((C6H8O3)n); PEO, plasma electrolytic oxidation; PLGA, poly(lactic acid-co-glycolic acid); QPEI, low-molecular-weight quaternized polyethyleneimine; RF-PECVD, Plasma Assisted Chemical Vapor Deposition; VAN, vancomycin; ZrN, zirconium nitride.

**Table 3:** Characteristics of studies that evaluated non-antibacterial surface treatment.

Author, year	Intervention		Comparison			Outcome – Antibacterial activity assay		Outcome – Cell viability assay		
	Groups	Surface treatment	Control	Essay	Bacteria	Result	Essay	Cell	Result	Conclusion
Geng et al., 2018 [32]	Ti Bacterial assay G1 = PBS (control) G2 = Ti/TBP-1-GGG/hBD3-1 G3 = Ti/TBP-1-GGG-hBD3-2 G4 = Ti/TBP-1-GGG-hBD3-3 Osteoblast assay G5 = blank Ti G4 = Ti/TBP-1-GGG-hBD3-3	TBP-1-mediated linkage	Bacterial assay PBS Osteoblast assay blank Ti	CFU	S. oralis, S. gordonii, and S. sanguinis	G4 > G2 > G3 > G1 p < 0.05 to G2	CLSM	MC3T3-E1	G4 did not exhibit significant cytotoxicity to MC3T3-E1 cells. It is noteworthy that the value of p was not informed.	G4 exhibited the non-cytotoxic antibacterial property
Godoy-Gallardo et al., 2016 [8]	Bacterial assay And Osteoblast assay Ti G1 = Ti G2 = Ti/ALCALIN G3 = TI/ALCALIN/TESPSA	Silane reactions	Bacterial assay Ti Osteoblast assay TCPS and Ti	CFU	S. sanguinis and L. salivarius	S. sanguinis: G3 > G2 > G1 L. salivarius: G3 > G2 > G1 Statistically significant differences between G3 and G1 in all readings (p < 0.05)	LDH Cytotoxicity Detection Kit	SaOS-2	G1>G2>G3>TCPS p < 0.05 to G3, G2 e G1 after 7 days	TESPSA immobilization on titanium promoted non-cytotoxic antibacterial activity
Zboun et al., 2022 [37]	Bacterial assay And Osteoblast assay Ti–6Al–4V G1 = Ti–6Al–4V/SLA G2 = Ti–6Al–4V/SBF G3 = Ti–6Al–4V/SBF–B G4 = Ti–6Al–4V/SAF	Acid etches and blasting.	Bacterial assay and Osteoblast assay No control group	CFU e CLSM	S. mutans e P. gingivalis	S. mutans G3>G2>G4>G1 P. gingivalis G3>G2>G1>G4 p < 0.0002 to both bacteria	CCK-8	hFOB	Cell viability in descending order. G3>G2>G1>G4 p = 0.0003	The proposed surfaces in G2 and G3 showed non-cytotoxic antibacterial activity. However, G3 showed better results than G2.
Zhou et al., 2019 [38]	Ti–6Al–4V G1 = 2 μm Micro (100 μm wide) G2 = 2 μm Micro (50 μm wide) G3 = 2 μm Micro (20 μm wide) G4 = 2 μm Micro (10 μm wide) G5 = 2 μm Micro (5 μm wide) G6 = 2 μm Micro+85 nm Nano (100 μm wide) G7 = 2 μm Micro+85 nm Nano (50 μm wide) G8 = 2 μm Micro+85 nm Nano (20 μm wide) G9 = 2 μm Micro+85 nm Nano (10 μm wide) G10 = 2 μm Micro+85 nm Nano (5 μm wide) G11 = 2 μm Micro +55 nm Nano (100 μm wide) G12 = 2 μm Micro +55 nm Nano (50 μm wide) G13 = 2 μm Micro +55 nm Nano (20 μm wide) G14 = 2 μm Micro +55 nm Nano (10 μm wide) G15 = 3.6 μm Micro+55 nm Nano (5 μm wide) G16 = 3.6 μm Micro+55 nm Nano (100 μm wide) G17 = 3.6 μm Micro+55 nm Nano (50 μm wide) G18 = 3.6 μm Micro+55 nm Nano (20 μm wide) G19 = 3.6 μm Micro+55 nm Nano (10 μm wide) G20 = 3.6 μm Micro+55 nm Nano (5 μm wide)	topographic	Bacterial assay and Osteoblast assay Pristine	CFU	P. gingivalis	2um micro G4>G5>G3>G2>G1 2um micro +85 nm Nano G9>G10 > G8>G6>G7 2um micro +55 nm Nano G14 > G15 > G13 > G12 > G11 3.6um micro +55 nm Nano G18 > G17 > G16 > G20 > G19 p < 0.05, n = 9	CCK-8	MG-63	2um micro G3>G4>G2>G5>G1 2um micro +85 nm Nano G10 > G9>G7>G6>G8 2um micro +55 nm Nano G11 > G13 > G14 > G15 > G12 3.6um micro +55 nm Nano G16 > G19 > G20 > G17 > G18 p < 0.05, n = 6	Surface modification with two μm deep and ten μm wide groove promoted non-cytotoxic antibacterial activity

CFU, colony forming units; CLSM, confocal laser scanning microscopy; hFOB, Human fetal osteoblastic cells LDH, lactate dehydrogenase; PBS, phosphate buffered saline; SAF, blasting with aluminum oxide and tetrafluoroboric acid and corrosion; SaOS-2, sarcoma osteogenic osteoblast-like cell line; SBF, sandblasting of aluminum oxide particles with boric acid and tetrafluoroboric acid etching; SBF–B, SBF + boric acid surface wetting SLA, Sandblasted acid-etched; TBP-1, TATA-binding protein associated factor 1; TBP-1-GGG-hBD3-1, chimeric peptide 1; TBP-1-GGG-hBD3-2, chimeric peptide 2; TBP-1-GGG-hBD3-3, chimeric peptide 3; TCPS, tissue culture polystyrene; TESPSA, triethoxysilylpropyl succinic anhydride.

The risk of bias was assessed independently by R.L.O.R and J.D.C.T through the adapted quasi-experimental studies appraisal tool by The Joanna Briggs Institute as previously performed by Gama et al., 2020 [52]. The classification of risk of bias was realized in accordance with answers to the questions. When answered "yes" for all the questions, the risk of bias was low (high methodological quality); when answered "yes" for 6 or 7 of the questions moderate risk of bias (moderate methodological quality), and five or less of the questions high risk of bias (low methodological quality).

## 3 Results

### 3.1 Selection process

**Fig. 1:**
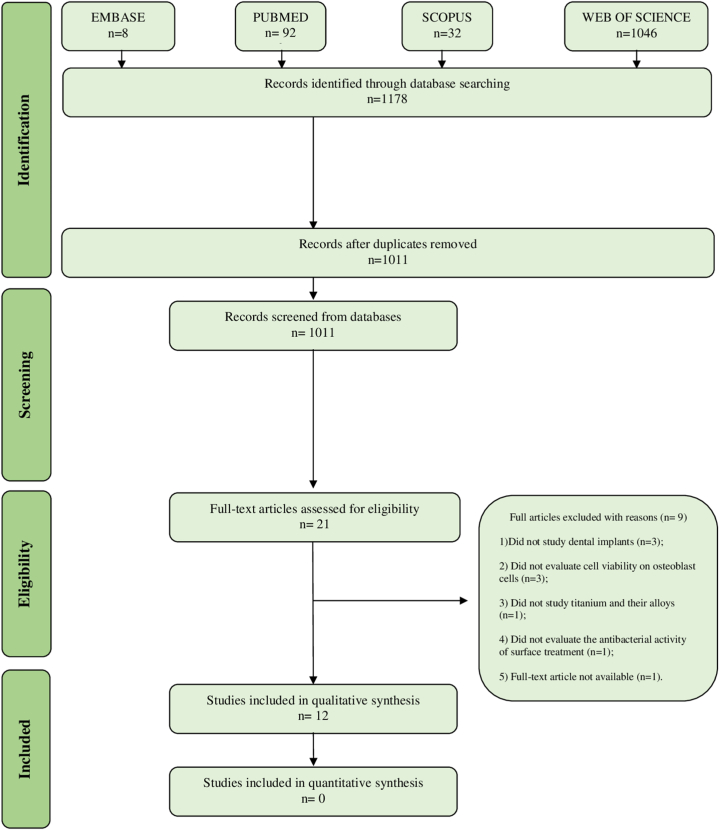
Flow diagram of literature search and selection criteria.

The search strategy found 1178 articles in the databases. After removing duplicates in EndNote Web, it resulted in 1011 articles to be evaluated by title and abstract, of which 21 were selected for reading in full, of these 12 [7,8,27,[30], [31], [32], [33], [34], [35], [36], [37], [38]] were included in this systematic review because they met the eligibility criteria and nine were excluded (Appendix 2). It is noteworthy that no article was included in the list of references and the gray literature (Fig. 1).

To facilitate understanding of the included studies, they were subdivided into groups according to the type of surface treatment applied.

### 3.2 Qualitative analysis of the included studies

#### 3.2.1 Antibacterial surface treatments

Table 2 shows the included studies that applied chitosan (CS) [9,27,35], poly (adipic anhydride) ((C6H8O3)n) (PADA) [7], and poly(lactic acid-co-glycolic acid) (PLGA) [33] associated with the antibiotic cephalexin (CEF) [7], vancomycin (VAN) [7], amoxicillin (AMX) [7]; CS polymer associated with an antibacterial material bioactive glass (58S-BG) [35], the addition of antibacterial silver ion (Ag) [34]; and addition of antibacterial materials such as zirconium nitride (ZrN) [36] and low-molecular-weight quaternized polyethyleneimine (QPEI) [31].

From the evaluated studies, it is observed that in the studies by Kazek-Kęsik et al., 2019 [33], Leśniak-Ziółkowska et al., 2020 [7], López-Ortega et al., 2022 [34], Mokhtari et al., 2018 [35], Norowski et al., 2011 [27], Pistilli et al., 2018 [36], antimicrobial surface treatments showed non-cytotoxic antibacterial activity for osteoblastic cells. With the exception of the studies from Song et al., 2022 [30]and Sun et al., 2019 [31] that the treatments were antibacterial but showed osteoblastic cytotoxicity.

#### 3.2.2 Non-antibacterial surface treatments

Table 3 shows the included studies that applied non-antibacterial surface treatment such as the addition of TBP-1-GGG-hBD3-(1, 2 and 3) [32] peptides, alkaline treatment, and addition of triethoxysilylpropyl succinic anhydride (TESPSA) [8], sandblasting [37], and creating roughness with micro and nano-scaled patterns [38]. From the evaluated studies, it was observed that all of them showed non-cytotoxic antibacterial activity for osteoblastic cells.

### 3.3 Meta-analysis

The quantitative synthesis could not be carried out due to the heterogeneity of the studies regarding the evaluated titanium alloy, surface treatment, antibacterial activity assay, bacteria, cell viability assay, and osteoblastic cell type.

### 3.4 Risk of bios

**Fig. 2:**
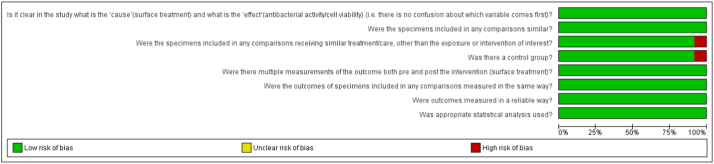
Qualitative analysis with adapted the quasi-experimental studies appraisal tool by the Joanna Briggs Institute.

**Fig. 3:**
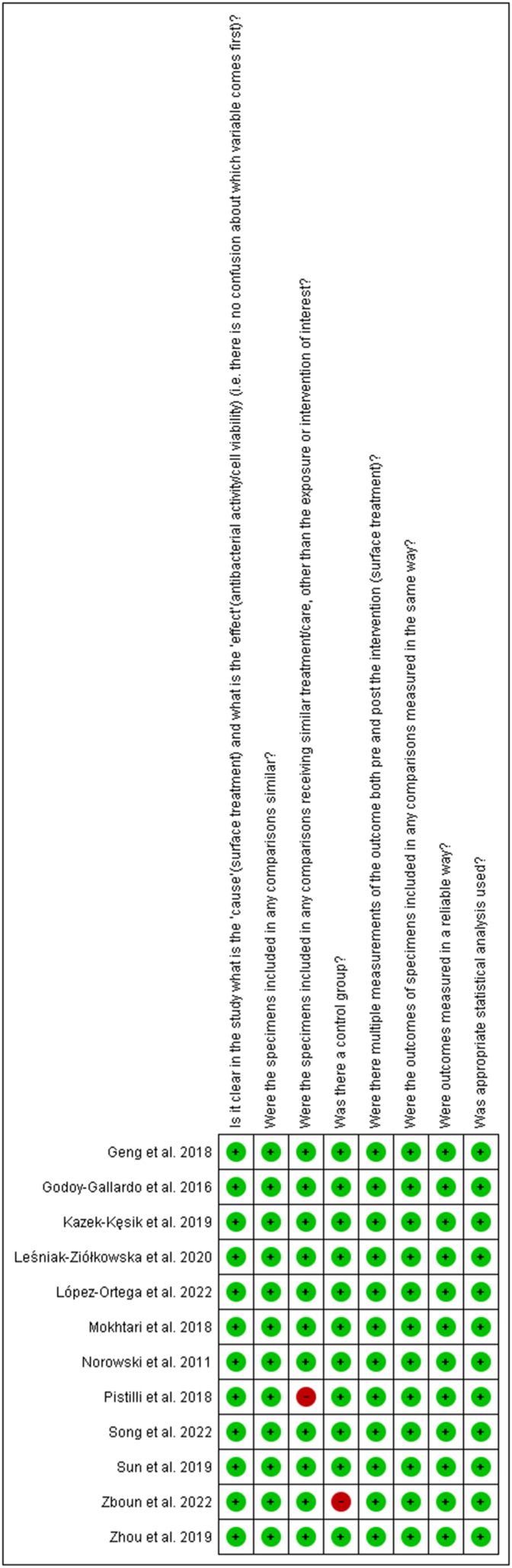
Qualitative analysis with adapted the quasi-experimental studies appraisal tool by the Joanna Briggs Institute per study.

The risk of bias assessment showed that ten studies were classified as having a low risk of bias and two studies as having a moderate risk of bias. The study by Zboun et al., 2022 [37] demonstrated increased levels of risk due to the lack of a control group, and Pistilli et al., 2018 [36] for not including similar surface treatments for comparisons (Fig. 2, Fig. 3).

## 4 Discussion

The inflammatory response to the pathogenic microbiota caused by peri-implant infections is associated with rates of 32% for the failure of dental implants and 73.2% for lack of osseointegration [1,2]. However, most surface treatments with antibacterial purposes performed on dental implants in order to remedy this gap have cytotoxic effects [31,53]. Therefore, to understand the antimicrobial mechanisms of surface treatments of titanium dental implants and their alloys and their effects both on bacterial activity and on cell viability of osteoblastic cells under simulated *in vitro* conditions, this systematic review was carried out with the aim of inferring the best non-cytotoxic antibacterial surface treatment for osteoblastic cells.

The studies that met the eligibility criteria were heterogeneous in relation to the applied surface treatment, titanium alloy, type of cell evaluated, and bacterial strains, which made it impossible to answer the research question and carry out the quantitative meta-analysis. Therefore, a thorough qualitative systematic review was performed to provide the best synthesis of the available literature [7,8,27,[30], [31], [32], [33], [34], [35], [36], [37], [38]].

Surface treatment with bioactive glass has been the subject of research in recent years and has become promising due to the indirect antibacterial action promoted by its chemical composition when interacting with the titanium dioxide layer of the implant [6,35,49]. These inferences are supported by Mokhtari et al., 2018 [35], who compared the application of the BG coating on different substrates (Ti, TNT/QS, and Ti/QS) and observed better rates of antibacterial activity in the TNT-BG group, attributed to the mechanical interaction between TNTs and BG providing a greater amount of QS on the surface. Furthermore, the increase in roughness and hydrophilicity provided by BG allowed a greater proliferation of osteoblastic cells.

Sun et al., 2019 [31] proposed the use of an antibacterial polymer, QPEI, and demonstrated that it presents good osteoblastic cell viability only when associated with ALEN, a drug that is biocompatible with osteoblasts and has high affinity with bone minerals. The antibacterial activity of QPI is attributed to bacterial cell membrane disruption. In addition, it has a low affinity for bacterial adhesion, attributed to the presence of hydroxyl groups in the formation process of this coating with broad-spectrum antibacterial and antifouling properties [54].

The studies of Mokhtari et al., 2018 [35] and Sun et al., 2019 [31] demonstrate that the chemical composition of nanomaterials, BG and QPEI, present molecular arrangements that promote low electrostatic affinity for bacterial cells and high affinity for osteoblastic cells. Thus, promising materials for the development of pro-osteoblastic antibacterial titanium implants.

Innovative local drug delivery systems for dental implants have been proposed to avoid side effects and antibacterial resistance when compared to oral therapy [55]. Kazek-Kęsik et al., 2019 [33] and Leśniak-Ziółkowska et al., 2020 [7] proposed the hybrid coating of polymers PLGA [33] and PADA [7] on Ti–15Mo and Ti–2Ta–3Zr–36Nb surfaces, respectively, treated with PEO associated with antibiotics AMX [7,33], CEF [7] e VAN [7]. The PEO treatment is proposed to be carried out prior to the addition of the polymeric coating, as it promotes an oxidized porous surface that improves the adhesion of the coating. In addition, the literature [7,33] reports that the porous morphology obtained by PEO improves osteoblastic cell viability.

Natural polymers such as CS are also associated with antibiotics and antimicrobials, as demonstrated by Song et al., 2022 [30] and Norowski et al., 2011 [27]. Norowski et al., 2011 [27] demonstrated that the evaluated antimicrobials (tetracycline and chlorhexidine) showed good results regarding their cytotoxicity and antibacterial activity. Tetracycline, when compared to chlorhexidine, presented a prolonged release time due to the electrostatic interaction with CS and did not present a temporary cytotoxic effect.

The non-cytotoxic antibacterial activity of polymeric hybrid coatings with antibiotics is attributed to the slow degradation capacity of the surface layers of polymers, which allows a slow release of antibiotics; this is a favorable characteristic for preventing bacterial adhesion and not inhibiting osteoblastic activity [7,33]. However, the authors of this review infer that it is challenging since more than one processing technique is required and the difficulty of establishing ideal concentrations of the drug in order not to be cytotoxic.

Zhou et al., 2019 [38], when preparing patterns of microgrooves with various widths and depths, they attributed the increase in cell viability and antibacterial activity to greater wettability resulting from the increase in depth and width of the microgrooves. Godoy-Gallardo et al., 2016 [8] corroborate this finding by also attributing the increase in antibacterial activity and cell viability to the increased wettability of surfaces treated with the alkaline treatment and the TEPSA silane. In addition, they point out that the presence of the TEPSA silane promoted an increased expression of BMP genes −2 and RUNX-2, responsible for inducing osteoblastic proliferation.

Zirconium nitride (ZrN) is a nanomaterial that has shown promise for limiting bacterial colonization compared to titanium nitride and other surface treatment approaches such as physical vapor deposition, thermal oxidation, and structuring with laser radiation [[56], [57], [58]]. Pistilli et al., 2018 [36] demonstrated that the ZrN coating bioactivated by argon plasma enables higher rates of osteoblastic adhesion and reduction of bacterial activity compared to smooth titanium because its external electronic surface promotes hydrophilicity.

According to the review by Gitens et al., 2014 [59], bacteria have an affinity for surfaces of the same hydrophilicity, so the authors of this review suggest based on studies of Zhou et al., 2019 [38], Godoy-Gallardo et al., 2016 and [8], Pistilli et al., 2018 [36] those surface treatments that promote hydrophilicity different from pathogenic bacteria can act as anti-adhesives; thus wettability being an important property to consider when developing antibacterial implants.

Bacterial adhesion inhibition can be avoided by the adsorption of chimeric peptides on the surface of titanium and its alloys [32,60]. Geng et al., 2018 [32] proposed hBD3 peptides, which are endogenous human antimicrobial peptides (AMPs) that have broad-spectrum antibacterial activity. Of the peptides tested, TBP-1-GGG-hBD3-3 showed better antibacterial activity than the other two peptides (TBP-1-GGG-hBD3-(1 and 2)), a factor attributed to its greater positive charge, as well as its possible ability to disrupt the bacterial cell membrane and inhibit RNA replication. These findings are promising, as the antibacterial capacity of these peptides can be extended to drug-resistant microbes, in addition to not being cytotoxic to osteoblastic cells, making them a promising approach for antibacterial dental implants.

An ion with excellent antibacterial properties is Ag, as it acts by destroying the bacterial cell wall. Tardelli et al., 2021 [16] inferred that the cytotoxic effect of the chemical element is dependent on 1) dose, 2) exposure time, 3) nanoparticle size, 4) average temperature, and 5) cell type. In this context, López-Ortega et al. [34] 2022 explored the antibacterial effects of incorporating Ag ions (biocidal agent) into Ti nanostructured surfaces by PEO in association with Ca and P ions (bioactive agents) due to their excellent osteoinductive properties. However, the presence of Ag in TNTs showed not only antimicrobial activity but also demonstrated increased levels of expression of osteogenic genes, a fact attributed to the titanium being implanted with Ag in a nanostructured surface. Thus, this study infers that the toxicity of antimicrobial ions such as Ag can be reduced when associated with other biocomposites, corroborating the literature [[41], [42], [43], [44],^61^,^62^].

On the other hand, Zboun et al., 2022 [37] promoted roughness in titanium surfaces by sandblasting and acid attack (boric acid). Osteoblastic differentiation and antibacterial activity were amplified in this study by the presence of boric acid boron compounds, which offer anti-osteoporotic and anti-inflammatory properties. However, the ideal concentrations of boron have not been clarified and, therefore, motivate future studies that quantify the minimum and maximum concentrations for promoting non-cytotoxic antibacterial activity.

The proposed systematic review, although it cannot answer the question that directed it due to the heterogeneity of the studies, allows inferring those surface treatments that alter the electrostatic condition are by the addition of polymers such as QPEI [31] or antibacterial nanomaterials such as BG [35] and CS [27,30,35] are promising because they allow osteoblastic cell viability and can reduce bacterial resistance rates and side effects when compared to oral antimicrobials and slow local release in polymeric coatings. Thus, electrostatic surface treatments are promising for regulating bacterial adhesion on titanium surfaces through long-range forces, as demonstrated by Kreve and Reis 2021 [40].

## 5 Conclusions

1) The literature surveyed in this systematic review, according to PRISMA standards, did not allow answering the question “which surface treatment for dental implants made of titanium and its alloys has the greatest non-cytotoxic antibacterial activity for osteoblastic cells?” due to the heterogeneity of the studies (titanium alloy, surface treatment, antibacterial test, strain, cell, and cell viability test).
2) Of the 12 studies evaluated in this systematic review, ten studies presented surface treatments with non-cytotoxic antibacterial activity. Meanwhile, the addition of calcium phosphate, chitosan, and cefazolin in pulsed current and QPEI in Ti were cytotoxic to osteoblastic cells despite being antibacterial.
3) Of the evaluated surface treatments, those that altered the electrostatic condition of the surface by adding nanomaterials, QPEI, BG, and CS, are considered promising because they reduce the chances of bacterial resistance by controlling their adhesion by electrical forces.

## Author contribution statement

Renan Rigotti, Juliana Dias Corpa Tardelli and Andrea Candido Reis: Conceived and designed the experiments; Performed the experiments; Analyzed and interpreted the data; Contributed reagents, materials, analysis tools or data; Wrote the paper.

## Funding statement

This research did not receive any specific grant from funding agencies in the public, commercial, or not-for-profit sectors.

## Data availability statement

Data included in article/supplementary material/referenced in article.

## Declaration of interest's statement

The authors declare no conflict of interest.

## Footnotes

Appendix A Supplementary data related to this article can be found at https://doi.org/10.1016/j.heliyon.2023.e13693.
