# Supplementary material for: Influence of antibacterial surface treatment on dental implants on cell viability: A systematic review
Source: Heliyon. 2023 Feb 16;9(3):e13693. doi: 10.1016/j.heliyon.2023.e13693 (PMC9988489; doi:10.1016/j.heliyon.2023.e13693)
Supplement: Multimedia component 1 [file mmc1.docx]

Appendix 1 - Database search strategy.

| Database | Search | Found |
| --- | --- | --- |
| EMBASE  November  17th, 2022 | ‘dental implant’ AND (titanium alloys OR titanium) AND ‘surface treatment’ AND (osteoblast OR ‘cytotoxicity’ OR ‘cell viability’) AND (bacteria OR ‘antibacterial activity’) | 8 |
| PubMed  November  17th, 2022 | ‘’dental implant’’ AND (titanium alloys OR titanium) AND ‘’surface treatment’’ AND (osteoblast OR ‘’cytotoxicity’’ OR “cell viability”) AND (bacteria OR “antibacterial activity’’) | 92 |
| Scopus  November  17th, 2022 | ‘’dental implant’’ AND (titanium alloys OR titanium) AND ‘’surface treatment’’ AND (osteoblast OR ‘’cytotoxicity’’ OR “cell viability”) AND (bacteria OR “antibacterial activity’’) | 32 |
| Science Direct  November  17th, 2022 | ‘’dental implant’’ AND (titanium alloys OR titanium) AND ‘’surface treatment’’ AND (osteoblast OR ‘’cytotoxicity’’ OR “cell viability”) AND (bacteria OR “antibacterial activity’’) | 1046 |
