# Supplementary material for: Influence of antibacterial surface treatment on dental implants on cell viability: A systematic review
Source: Heliyon. 2023 Feb 16;9(3):e13693. doi: 10.1016/j.heliyon.2023.e13693 (PMC9988489; doi:10.1016/j.heliyon.2023.e13693)
Supplement: Multimedia component 2 [file mmc2.docx]

Appendix 2. Excluded articles and reasons for exclusion.

| Author, Year | Reason for exclusion |
| --- | --- |
| Thukkaram et al. 2021(1) | 1 |
| Costa et al. 2020(2) | 2 |
| Zhang et al. 2020(3) | 1 |
| Shao et al. 2020(4) | 1 |
| Lu et al. 2020(5) | 2 |
| Sato et al. 2020(6) | 3 |
| Wu et al. 2019(7) | 4 |
| Hatoko et al. 2019(8) | 2 |
| Sun et al. 2018(9) | 5 |

1)Did not study dental implants (n=3), 2) Did not evaluate cell viability on osteoblast cells (n=3), 3) Did not study titanium and their alloys (n=1), 4) Did not evaluate the antibacterial activity of surface treatment (n=1), 5) Full-text article not available (n=1).

REFERENCES

1. Thukkaram M, Vaidulych M, Kylián O, Rigole P, Aliakbarshirazi S, Asadian M, et al. Biological activity and antimicrobial property of Cu/a-C: H nanocomposites and nanolayered coatings on titanium substrates. Mater Sci Eng C [Internet]. 2021;119(May 2020):111513. Available from: https://doi.org/10.1016/j.msec.2020.111513

2. Costa RC, Souza JGS, Cordeiro JM, Bertolini M, de Avila ED, Landers R, et al. Synthesis of bioactive glass-based coating by plasma electrolytic oxidation: Untangling a new deposition pathway toward titanium implant surfaces. J Colloid Interface Sci [Internet]. 2020;579:680–98. Available from: https://doi.org/10.1016/j.jcis.2020.06.102

3. Zhang X, Huang Y, Wang B, Chang X, Yang H, Lan J, et al. A functionalized Sm/Sr doped TiO2 nanotube array on titanium implant enables exceptional bone-implant integration and also self-antibacterial activity. Ceram Int [Internet]. 2020;46(10):14796–807. Available from: https://doi.org/10.1016/j.ceramint.2020.03.004

4. Shao S-Y, Chen J-X, Tang H-Y, Ming P-P, Yang J, Zhu W-Q, et al. A titanium surface modified with zinc-containing nanowires: Enhancing biocompatibility and antibacterial property in vitro. Appl Surf Sci. 2020;515.

5. Lu X, Xiong S, Chen Y, Zhao F, Hu Y, Guo Y, et al. Effects of statherin on the biological properties of titanium metals subjected to different surface modification. Colloids Surfaces B Biointerfaces. 2020;188(October 2019).

6. Sato PS, Watanabe T, Maeda H, Obata A, Kasuga T. Preparation of an antibacterial amorphous thin film by radiofrequency magnetron sputtering using a 65ZnO–30P2O5–5Nb2O5 glass. J Non Cryst Solids [Internet]. 2020;528(July 2019):119724. Available from: https://doi.org/10.1016/j.jnoncrysol.2019.119724

7. Wu H, Xie L, He M, Zhang R, Tian Y, Liu S, et al. A wear-resistant TiO2 nanoceramic coating on titanium implants for visible-light photocatalytic removal of organic residues. Acta Biomater [Internet]. 2019;97:597–607. Available from: https://doi.org/10.1016/j.actbio.2019.08.009

8. Hatoko M, Komasa S, Zhang H, Sekino T, Okazaki J. UV treatment improves the biocompatibility and antibacterial properties of crystallized nanostructured titanium surface. Int J Mol Sci. 2019;20(23).

9. Sun FQ, Li MQ, Peng SH, Zhang HM, Liu M, Qu XY. [Study on antibacterial properties and osteoblast activity of antimicrobial peptide coatings on titanium implants]. Zhonghua kou qiang yi xue za zhi = Zhonghua kouqiang yixue zazhi = Chinese J Stomatol. 2018 Jun;53(6):419–24.
